# The impact of aminopyrene trisulfonate (APTS) label in acceptor glycan substrates for profiling plant pectin β-galactosyltransferase activities

**DOI:** 10.1016/j.carres.2016.07.017

**Published:** 2016-10-04

**Authors:** Stephan Goetz, Martin Rejzek, Sergey A. Nepogodiev, Robert A. Field

**Affiliations:** Department of Biological Chemistry, John Innes Centre, Norwich Research Park, Norwich NR4 7UH, UK

**Keywords:** Galactosyltransferase, Pectin, Acceptor substrate, Fluorophore, Capillary electrophoresis

## Abstract

Aminopyrene trisulfonate (APTS)-labelled disaccharides are demonstrated to serve as readily accessible acceptor substrates for galactosyltransferase activities present in Arabidopsis microsome preparations. The reductive amination procedure used to install the fluorophore results in loss of the ring structure of the reducing terminal sugar unit, such that a single intact sugar ring is present, attached via an alditol tether to the aminopyrene fluorophore. The configuration of the alditol portion of the labelled acceptor, as well as the position of alditol galactosylation, substantially influence the ability of compounds to serve as Arabidopsis galactosyltransferase acceptor substrates. The APTS label exhibits an unexpected reaction-promoting effect that is not evident for structurally similar sulfonated aromatic fluorophores ANDS and ANTS. When APTS-labelled β-(1 → 4)-Gal_3_ was employed as an acceptor substrate with Arabidopsis microsomes, glycan extension generated β-(1 → 4)-galactan chains running to beyond 60 galactose residues. These studies demonstrate the potential of even very short glycan-APTS probes for assessing plant galactosyltransferase activities and the suitability CE-LIF for CAZyme profiling.

## Highlights

• APTS-labelled disaccharides serve as acceptor substrates for galactosyltransferases.
• Configuration of the alditol linker and site of glycosylation influence GalT turnover.
• APTS shows a reaction-promoting effect not evident for similar fluorophores.
• β-(1 → 4)-Gal_3_-APTS acceptor supports enzymatic extension to > 60 galactose residues
• Demonstrates the potential of glycan-APTS probes with CE-LIF for CAZyme profiling.

## Graphical abstract

**Figure:**
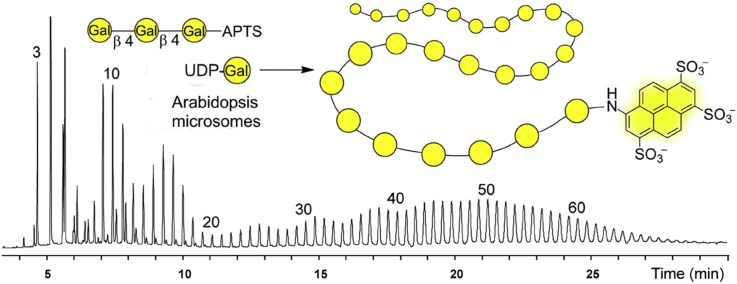

## 1 Introduction

**Fig. 1:**
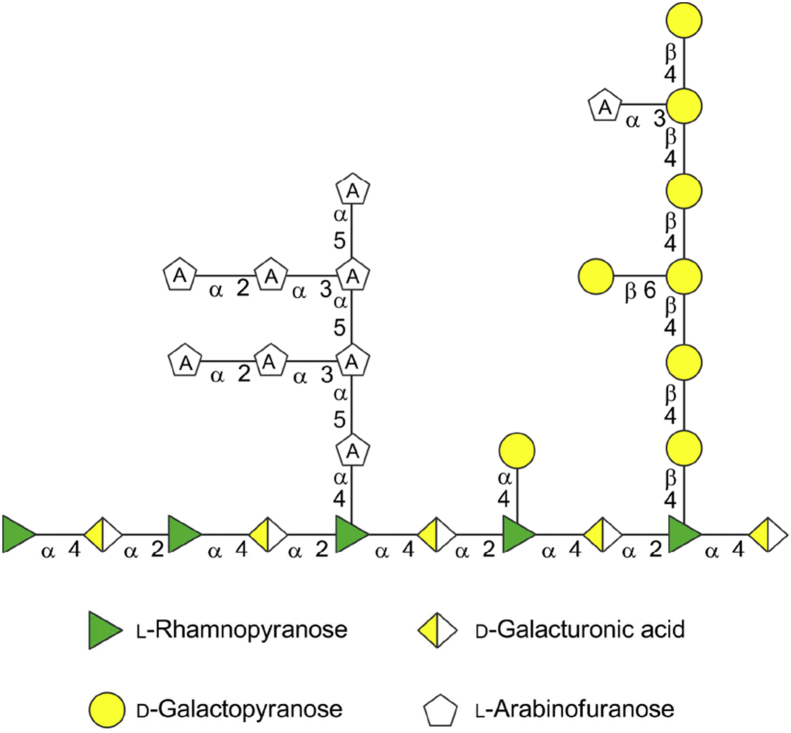
Schematic representation of RG-I region of the complex multi-component pectin structure (adapted from Somerville et al. [6]). Side chain galactans are composed of β-(1 → 4)-linked galactosyl residues which form the backbone structure which can be further decorated with β-(1,6)-linked galactosyl residues to introduce branching points or α-(1 → 3)- and α-(1 → 5)-linked Ara*f* residues.

Pectins are structurally and functionally amongst the most complex polysaccharides [1] and they have versatile functions in plant morphology, growth, development, cell adhesion and plant defence. Pectins and their derivatives also find many applications [2], especially in the food industry as gelling and stabilizing agents. Current research also suggests potential biomedical use for pectins, since it has been shown that they can stimulate the human immune response, lower cholesterol and serum glucose levels, as well as have beneficial effects in cancer [3]. The ability to manipulate pectin structure at the whole plant level therefore has implications for both fundamental science and application. While many advances have been made in the past decade, detailed understanding of the enzymes required for pectin biosynthesis is still at best patchy [4]. With this in mind, we have considered the development of fluorescent glycan probes suitable for the analysis of plant cell wall glycosyltransferase activities [5]. In the current study we report on galactoside-based probes as acceptor substrates for biosynthesis studies on pectic galactan (Fig. 1). This galactan is a linear polymer composed of β-(1 → 4)-linked galactopyranose residues attached via its reducing end to the rhamnopyranose residue of rhamnogalacturonan-I (RG-I) which is one of the pectin backbone polysaccharides.

It is not known how pectic galactan is synthesised in detail, although it has been suggested that three groups of membrane-bound galactosyltransferases (GalTs I, II and III) are involved in the biosynthesis process [7]. Group I enzymes are assumed to transfer a galactosyl residue onto a rhamnosyl residue of the RG-I backbone, thereby initiating the galactan side-chain. The transfer of a second galactosyl residue to form the first Gal-β-(1 → 4)-Gal linkage is proposed to be catalysed by group II enzymes. In the last step, a group III enzyme(s) transfer one or more galactosyl residues to elongate the galactan side-chain. Factors that determine chain length of polymerised galactan remain unknown. There are currently no reports of group I GalT enzyme activities that initiate galactan biosynthesis directly on the RG-I backbone. Peugnet and co-workers solubilised a flax microsomal GalT that was able to catalyse transfer of β-galactosyl residues onto RG-I short branches of β-(1 → 4)-galactan side chains [8]. The authors suggested that this specific GalT belongs to the group II enzymes which requires β-Gal(1 → 4)Rha as an acceptor substrate. However the detailed structure of the acceptor substrate, which in that case was a large polysaccharide RG-I with short branches, has not been determined. A similar group II activity was found in potato microsomes [9]. A polymerising β-(1 → 4)-galactan:β-(1 → 4)-galactosyltransferase activity was first described in 1973 in work with microsomes from *Vigna radiata* (mung bean) [10], with subsequent years seeing numerous reports of such transferases, including from *Linum usitassimum* (flax) [11], *Solanum tuberosum* (potato) [12], *Glycine max* (soybean) [13] and *Pinus radiata* (Monterey pine) [14]. More recently, Liwanag et al. reported the identification of the β-(1 → 4)-galactosyltransferase GALS1 from *Arabidopsis thaliana*. The enzyme was successfully transiently expressed in *Nicotiana benthamiana* leaves and it was shown that it could transfer up to four galactosyl residues onto a synthetic β-(1 → 4)-linked galactopentaose acceptor substrate [15].

More precise analysis of the enzymatic process behind β-(1 → 4)-galactan polymerisation has been achieved by the use of structurally defined, fluorescent 2-aminobenzamide-labelled (2-AB-labelled) acceptor substrates in conjunction with *V. radiata* microsomes [16]. The authors described a group III polymerising β-(1 → 4)-GalT activity, which required at least 4 galactose units (i.e. 2-AB-labelled galactotetraose) to initiate galactan polymerisation; 2-AB labelled galactotriose did not act as acceptor. Similar observations regarding GalT III acceptor substrate chain length preference were found with *G. max* microsomes [17]. Taken together, these data suggest a potential common preference of the Type III GalT enzymes for acceptor substrates of four or more galactose units. Given that such galactans, and other oligosaccharides of this size, are not readily available from nature, but are beginning to yield to chemical syntheses [18], [19], [20], we set out to explore the potential of simpler glycans. Anticipating a low turnover, we opted for high resolution, high sensitivity assays [5] based on 8-aminopyrene-1,3,5-trisulfonate-labelled (APTS-labelled) acceptor glycans with capillary electrophoresis coupled to laser-induced fluorescence detection (CE-LIF) [21], [22]. Similar approaches have been translated onto standard DNA sequencer [23], [24], [25], enabling much higher throughput of glycan structural analyses, which may also have potential for the high throughput profiling of carbohydrate-active enzymes.

## 2 Experimental

### 2.1 General

Protein concentrations were determined with detergent-compatible BrafordUltra reagent (Expedeon Ltd.) according to the manufacturer's instructions with a microplate reader at 595 nm. Fluorescent labels 8-aminopyrene-1,3,6-trisulfonic acid (APTS) (ThermoFisher Scientific), 8-aminonaphthalene-1,3,6-trisulfonic acid (ANTS) (ThermoFisher Scientific) and 7-aminonaphthalene-1,3-disulfonic acid (ANDS) (Sigma) were used as purchased. Authentic Gal-β-(1,6)-Gal was synthesised essentially as described in the literature [26].

### 2.2 Plant material

Suspension cultures of *Arabidopsis thaliana* (ecotype *Landsberg erecta*) were grown in 200 mL Erlenmeyer flask in the dark with constant agitation (23 °C, 110 rpm). Sterile conditions were maintained during cultivation. The media used for the cultivation consisted of Murashige and Skoog medium (Sigma) (4.4 g/L), sucrose (30.0 g/L), kinetin (0.05 mg/L), 1-naphthaleneacetic acid (0.5 mg/L) at pH to 5.8 adjusted with 1 M NaOH. Mung Bean (*Vigna radiata*) seeds were washed with tap water and placed in a tray on wetted tissues for germination. The hypocotyls were harvested 2–3 days after germination.

### 2.3 Isolation of microsomes

The protocol for the isolation of microsomes, from both mung bean and Arabidopsis, was adapted from the literature [27]. All isolation steps were carried out at 4 °C.

Mung bean hypocotyls were homogenised with a standard kitchen onion chopper (Argos) in homogenisation buffer (30% (v/v) glycerol and 1 mM DTT in 25 mM MES-KOH pH 6.5). The homogenised material was further homogenised in a mortar with pestle with dH_2_O-washed sea sand. The resulting slurry was filtered through two layers of Miracloth (Merck Chemicals) to give a suspension of cultured cells. The cells were harvested under vacuum on a paper filter disk and washed three times with 25 mM MES-KOH pH 6.5.

Mung bean or Arabidopsis cells were disrupted using a cell disruptor (Constant Systems Limited) and centrifuged for 10 min at 3000 × g. The supernatant was carefully layered onto a glycerol cushion (80% glycerol (v/v) in 25 mM MES-KOH pH 6.5) in ultracentrifuge tubes and further centrifuged at 100,000 × g for 1 h. The interface between the two layers contained microsomes, which were isolated with a Pasteur pipette. The microsomal fraction was diluted with four volumes of 25 mM MES-KOH pH 6.5 buffer and further centrifuged at 100,000 g for 1 h. The resulting pellet was homogenised with a Dounce tissue grinder in homogenisation buffer (without DTT) and stored at −80 °C.

### 2.4 Preparation of β-(1 → 4)-galacto-oligosaccharide acceptor substrates

A set of β-(1 → 4)-galacto-oligosaccharide acceptor substrates were prepared, each at milligram scale, by enzymatic partial digestion of potato β-(1 → 4)-galactan, followed by purification on Celite-charcoal [28]. Potato galactan (100 mg, Megazyme) was dissolved in H_2_O (10 mL) and added to 0.1 M NaOAc buffer pH 4.2 (10 mL). To this solution was added 20 U of *Aspergillus niger endo*-β-(1 → 4)-galactanase (Megazyme) and the mixture was incubated for 8 min at 40 °C. After incubation, the reaction was terminated by heating at 100 °C for 10 min. After cooling the solution was applied to an H_2_O-equilibrated anion exchange column (5 mL) filled with DEAE Sephadex A-50 (GE Healthcare) and the column was eluted with H_2_O (50 mL). The flow-through was concentrated with a rotary evaporator to a final volume of approximately 2 mL and the residue was purified on charcoal-Celite (1:1, w/w) column [28]. A column containing 1 g of the sorbent was loaded with 0.5 mL of material and eluted with a stepwise (50 mL) gradient of MeCN (0–12%) in H_2_O, giving fractions enriched with particular galacto-oligosaccharide which were concentrated with a rotary evaporator and re-dissolved in H_2_O (0.5 mL). Purity of products was confirmed by CE-LIF (Fig. S5 in SI); following derivatisation with APTS, as outlined below, fluorescent glycans were also characterised by mass spectrometry (Table S1 in SI).

### 2.5 Fluorescent labelling of glycans

A published procedure [29] for glycan labelling was adapted as follows: A sample of dried glycan (5 nmol) was dissolved in a solution prepared by mixing 5 μL of 0.2 M solution of fluorescent label (APTS or ANTS or ANDS) in 15% (v/v) AcOH and 5 μL of 1 M NaBH_3_CN in THF (prepared immediately prior to the reaction). The reaction was carried out in 0.2 mL PCR tubes (Starlab) and incubated at 37 °C for at least 15 h. After incubation the mixture was diluted with H_2_O and subjected to separation with FACE as described in section 2.7 but with 0.1 M Tris-HCl pH 8.2 as both the gel buffer and the running buffer. After separation, the gel cassette was opened and washed with H_2_O to remove unlabelled glycans. Fluorescent bands corresponding to the APTS-labelled oligosaccharides were excised from the gel and transferred into screw-cap reaction tubes together with H_2_O and a ceramic ball. The gel slices were homogenised to fine slurry with a FastPrep homogenizer (Thermo Savant), the slurry was extracted with H_2_O and centrifuged (10 min at 3000 × g) in 15 mL screw-cap centrifugation tubes (Becton Dickinson) to separate solid polyacrylamide. The extraction of solid residue was repeated several times, combined aqueous extracts (approximately 15 mL) were passed through Dowex 50WX8-400 (Na^+^ form, 2.5 mL) and the resin was eluted with H_2_O (10 mL) to give a solution of labelled glycan which was concentrated with a rotary evaporator.

### 2.6 Quantitation of fluorescently-labelled glycans

Concentrations of purified labelled glycans were determined in 50 mM bicarbonate buffer pH 8.5 using the molar extinction coefficient for APTS- and ANTS-labelled maltoheptaose (*ɛ*_450_ 17,160 M^−1^ cm^−1^ and *ɛ*_367_ 7050 M^−1^ cm^−1^ respectively) [21]. Since no published data are available for molar extinction of ANDS-carbohydrate adducts, the concentration of the ANDS derivative (absorption maximum at 256 nm) was determined using the molar extinction coefficient of the free fluorophore (*ɛ*_247_ = 3100 M^−1^ cm^−1^) [30].

### 2.7 Fluorophore-assisted carbohydrate electrophoresis (FACE) with polyacrylamide slab gels

Carbohydrates labelled with ANTS, APTS or ANDS were separated and analysed with polyacrylamide gels according to the published methodology [31]. For electrophoretic separation, a water-cooled electrophoresis system SE260 (Hoefer) was used. The gels were hand-cast between Mini-PROTEAN glass plates (Bio-Rad Ltd.). Typical gels were 8 × 7 cm and 1.0 or 1.5 mm thick. Fluorescently-labelled carbohydrate samples were mixed with glycerol prior to loading on the gel. The separation was carried out at constant 300 V and the run was monitored under UV-light to ensure efficient separation of individual bands. Separation gel: 20% (w/v) polyacrylamide gel and 0.5% (w/v) *N,N′*-methylenebisacrylamide in 0.1 M Tris adjusted to pH 8.2 with boric acid. Stacking gel: 8% (w/v) polyacrylamide gel and 0.2% (w/v) *N,N′*-methylenebisacrylamide in 0.1 M Tris adjusted to pH 8.2 with boric acid. Separation buffer: 0.1 M Tris adjusted to pH 8.2 with boric acid. After separation, the gel cassettes were removed from the electrophoresis chamber and visualised with a Fuji FLA-7000 imaging system equipped with the filter set for CY2 detection (473 nm excitation, 530 nm emission filter, Fuji Photo Film Co. Ltd).

### 2.8 Fluorophore-assisted carbohydrate analysis using capillary electrophoresis with laser-induced fluorescence detection (CE-LIF)

APTS-labelled glycans were analysed using a ProteomeLab PA800 CE instrument (Beckman Coulter) equipped with a LIF detection system operating at 488 nm excitation and 520 nm emission specifically designed for APTS-labelled glycan analysis. For the separation, a polyvinyl-coated N–CHO capillary (50.2 cm × 50 μm I.D., length to detector 40 cm, Beckman Coulter). Pressure-injection (0.5 psi, 20 s) was used to load the sample into the capillary. Samples were separated at 30 kV in 25 mM LiOAc-0.4% polyethylene oxide running buffer at pH 4.75 [32] for 7–45 min. Data were analysed with PA-800 plus software.

### 2.9 Galactosyltransferase assays

A typical glycosyltransferase reaction contained 25–100 μg microsomal protein, 10–100 μM APTS-labelled acceptor oligosaccharide, 0.5–1.0 mM UDP-galactose, 15 mM MnCl_2_, 25 mM MES-KOH buffer pH 6.5 and Triton X-100 (detergent/protein ratio 5:1) in a total volume of 50–100 μL. The reactions were incubated at room temperature for approximately 24 h. After incubation, the reactions were boiled for 5 min and centrifuged for 5 min at 11,000 × g. The supernatant was removed and used for analysis.

## 3 Results and discussion

### 3.1 Establishing conditions for microsomal GalT reactions

**Fig. 2:**
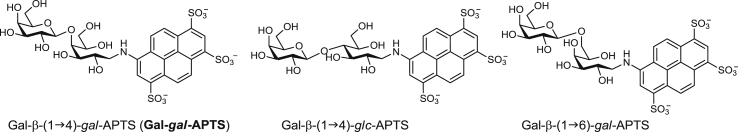
APTS-labelled β-Gal-containing acceptors derived from disaccharides Gal-β-(1 → 4)-Gal, Gal-β-(1 → 4)-Glc and Gal-β-(1 → 6)-Gal. Acronyms gal and glc denote galactitol and glucitol, respectively.

For batch to batch consistency, we opted to employ microsomal preparations from cultured Arabidopsis cells as a source of galactosyltransferase activities. To optimise conditions for microsomal GalT-catalysed reactions, a CE-LIF-based quantitative analysis was established for which basic principles were adapted from published research [16]. The assay followed the conversion of Gal-β-(1 → 4)-Gal-β-(1 → 4)-*gal*-APTS (**Gal**_**2**_**-*gal*-APTS**) into Gal-β-(1 → 4)-Gal-β-(1 → 4)-Gal-β-(1 → 4)-*gal*-APTS (**Gal**_**3**_**-*gal*-APTS,** see legend to Fig. 2) under the action of UDP-Gal and GalT in the presence of Triton X-100 at 20 °C. In order to facilitate simple end-point kinetic analyses, reactions were allowed to proceed to a maximum of 15% yield of **Gal**_**3**_**-gal-APTS** (ca. 6 h) when formation of longer oligomers was negligible (<2%). Thus it was established that GalT is active over a broad range of pH and Mn^2+^ concentrations, with maximal activity at pH 6 in the presence of 15–20 mM Mn^2+^ (SI Fig. S1A, S1B). Notably, the GalT was found to tolerate a broad range of detergent/protein ratios and detergent concentrations (SI Fig. S1C). Throughout the following series of experiments, 0.5% Triton X-100 was used at a detergent/protein ratio of 5:1. For optimal GalT activity the detergent needed to be peroxide-free and used fresh.

### 3.2 Impact of the reducing terminal sugar unit of disaccharide acceptors on galactosyltransferase activity

CE-LIF analysis of glycans requires their derivatisation via reductive amination with the fluorophore APTS. In the case of APTS-labelled glycans the ring of the reducing terminal sugar was transformed into an aminoalditol which serves as a tether between the residual glycan chain and the APTS label. The length, site of substitution, stereochemistry and conformation of this tether could potentially impact on the substrate reactivity. We therefore initially evaluated three isomeric APTS-labelled β-galactosylated compounds (Fig. 2), derived from disaccharides Gal-β-(1 → 4)-Gal, Gal-β-(1 → 4)-Glc (lactose) and Gal-β-(1 → 6)-Gal, as substrate acceptors for UDP-Gal with GalT. Analyses of the composition of mixtures of fluorescently-labelled glycans formed in these reactions were also used to assess galactosyltransferase activities in microsomes isolated from *A. thaliana* suspension cultured cells.

**Fig. 3:**
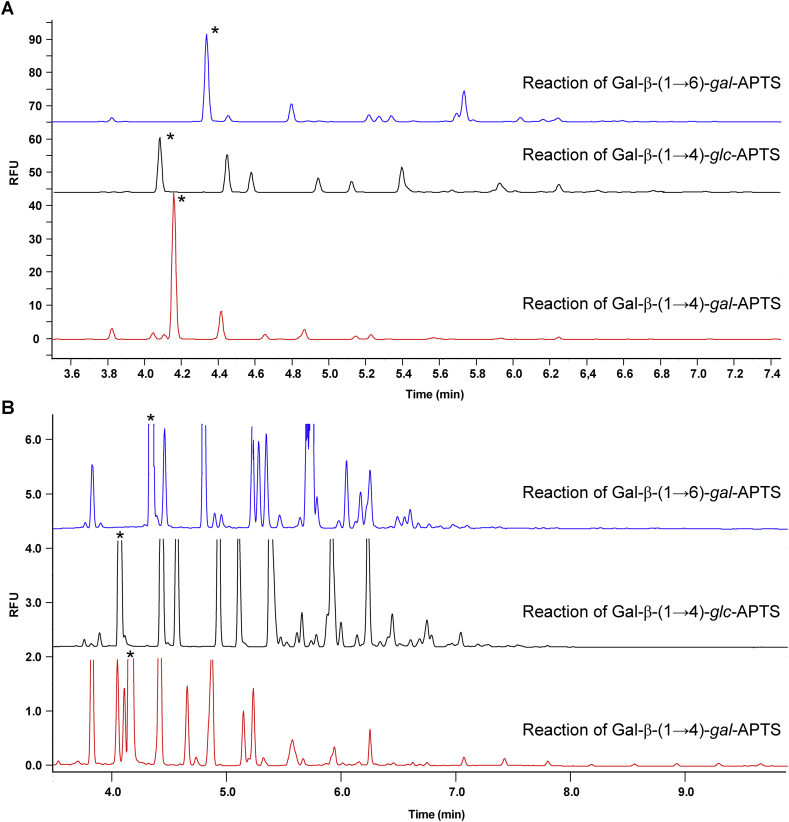
CE-LIF traces of the GalT assay with APTS-labelled disaccharides (peaks marked with an asterisk correspond to starting disaccharides) and UDP-Gal. **A**, Full scale and **B**, zoomed-in views. The assays were performed for 2 days at 20 °C with 100 μg of microsomal protein in 1.25% Triton X-100 (detergent/protein ratio 5:1), 25 mM Mes-KOH buffer pH 6.5 with 15 mM MnCl_2_, 200 μM APTS-labelled acceptor, 2.5% glycerol and 0.5 mM UDP-Gal.

The resolving power of CE enabled analyses of enzyme assays with the three isomeric Gal-containing acceptors (Fig. 3), which revealed that these rather minimal models of β-galactan are all acted upon by Arabidopsis galactosyltransferases, albeit at extended time (CE-LIF data show results after 48 h incubation). The efficiencies of acceptor substrate conversion and products distributions are different in each case. If homo-oligomerisation takes place, then CE-LIF would be expected to exhibit a series of regularly separated peaks, which is clearly not the case for major products formed from any of these three acceptors investigated. The lack of regularity in the CE-LIF electropherograms is indicative of the formation of more than one linkage type during the extension of these acceptor substrates. Assays with Gal-β-(1 → 4)-*gal*-APTS showed relatively low turnover of the substrate, but minor amounts of polymerised products were detectable up to degree of polymerisation (DP) ∼15. In contrast, a range of seven major products with >50% overall conversion of the acceptor substrate was found when a similar reaction was performed with Gal-β-(1 → 4)-*glc*-APTS. Similar to this result, reactions with Gal-β-(1 → 6)-*gal*-APTS contained a range of different, irregularly distributed products. Overall these experiments demonstrated that even simple monosaccharide structures tethered to APTS can indeed serve as competent plant galactosyltransferase acceptor substrates. However, the configuration of alditol portion of the APTS-labelled acceptor as well as the position of alditol galactosylation substantially influence the enzymatic reaction.

### 3.3 exo-Glycosidase digestion confirms formation of β-(1 → 4)-linkages

**Fig. 4:**
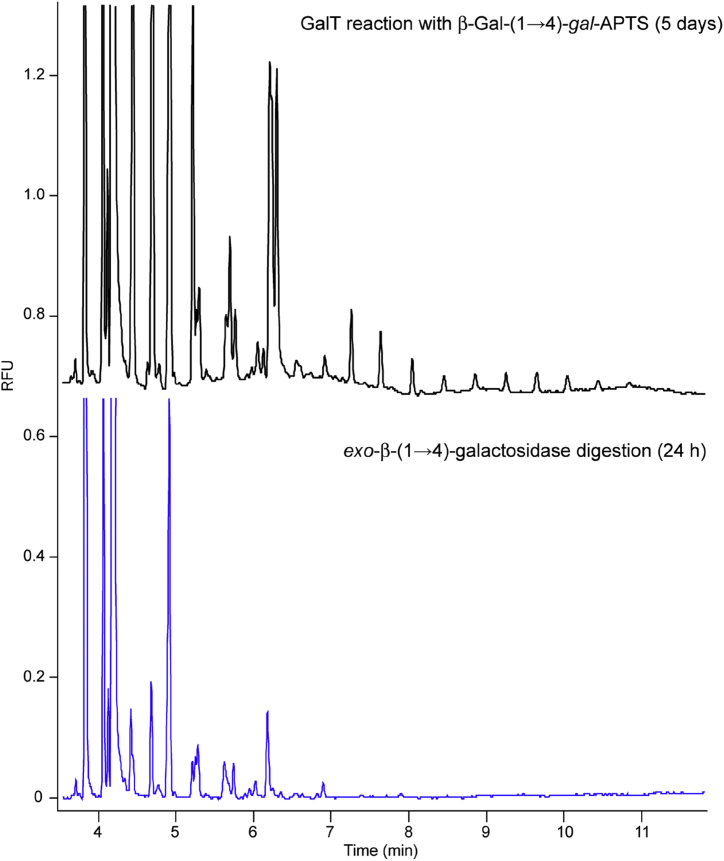
Digestion of the GalT reaction products with *exo*-β-(1 → 4)-galactosidase. Black trace: CE-LIF electropherogram of the products of galactan elongation after incubation of Gal-β-(1 → 4)-*gal*-APTS with GalT and UDP-Gal. Blue trace: CE-LIF analysis of the digestion of elongation products with *exo*-β-(1 → 4)-galactosidase. The galactosyltransferase reaction was performed for 5 days at 20 °C with 100 μg of microsomal protein in 1.25% Triton X-100 (detergent/protein ratio 5:1), 25 mM Mes-KOH buffer pH 6.5 with 15 mM MnCl_2_, 100 μM Gal-gal-APTS, 2.5% glycerol and 0.5 mM UDP-Gal. Enzymatic digestion was performed with 2 μU of *exo*-β-(1 → 4)-galactosidase (*Streptococcus pneumonia*e) for 24 h.

Detailed examination of the electropherogram originated from the reaction of Gal-β-(1 → 4)-*gal*-APTS revealed the presence of peaks with retention time between 7 and 10 min (Fig. 3B). When the same reaction was carried out for 5 days these products gave even more distinct evenly spaced peaks in 7–10 min region of CE-LIF trace (Fig. 4, more details in Fig. S3 in SI). Such regularity of appearances of CE signals may indicate that the products are homopolymers which originate from the action of a β-(1 → 4)-polymerising galactosyltransferase. However, clusters of poorly resolved peaks with shorter retention times (4–6.5 min) were also present suggesting possible formation of other types of inter-galactosidic linkages, most likely β-(1 → 3)- and/or β-(1 → 6)-linkages which are known to be present in plant galactans [33]. To verify identities of CE-LIF peaks the reaction mixture obtained from Gal-β-(1 → 4)-*gal*-APTS was treated with *exo*-β-(1 → 4)-galactosidase (*Streptococcus pneumoniae*). This digestion analysis revealed that most of the peaks in the electropherogram disappeared confirming that corresponding reaction products possessed β-(1 → 4)-linkages between galactose units and therefore synthesised by a β-1,4-galactan:β-1,4-galactosyltransferase. Several peaks remained unchanged (Fig. 4) revealing products resistant to β-(1 → 4)-galactosidase digestion. These products are likely to incorporate β-(1 → 3)- or β-(1 → 6)-linked galactose residues located at or close to the non-reducing terminus and blocking the action of the *exo*-β-(1 → 4)-galactosidase.

### 3.4 Galactosyltransferase reactions with unlabelled disaccharide acceptors

In a series of parallel experiments we examined the effect of the presence of the APTS residue in the acceptor substrates on the galactosyltransferase reactions. For this purpose, unlabelled disaccharides Gal-β-(1 → 4)-Gal, Gal-β-(1 → 4)-Glc and Gal-β-(1 → 6)-Gal were used as acceptors. Following incubation with microsomes and subsequent removal of protein by heat-mediated precipitation, each reaction mixture was subjected to APTS labelling and CE-LIF analysis. No products identical to those observed in the reactions with APTS-labelled acceptors were detected; only peaks originating from the non-elongated acceptors were detectable, along with some APTS-derived impurities (Fig. S4 in SI). We note that the use of even monosaccharide reducing sugar α-L-fucose has been used to good effect as an acceptor substrate in studies on α-(1,3)-xylosyltransferases involved in rhamnogalacturonan-II biosynthesis; however, α-L-fucose needed to be used at very high concentration indeed (500 mM) to achieve turnover [34].

### 3.5 Galactosyltransferase assays of APTS-labelled acceptors derived from galacto-oligosaccharides with DP 3-5

It should be noted that the turnover observed for Gal-β-(1 → 4)-*gal*-APTS was less than a few percent, even after extended incubation. This prompted us to move on to explore larger β-(1 → 4)-galactoside-terminating glycans in the search for more efficient substrates. To this end, we prepared four further APTS-labelled galacto-oligosaccharides: β-(1 → 4)-*galacto*-triose, -tetraose, -pentaose and –hexaose (characterisation data can be found in the Supporting Information Table S1, Figs. S8–S14).

**Fig. 5:**
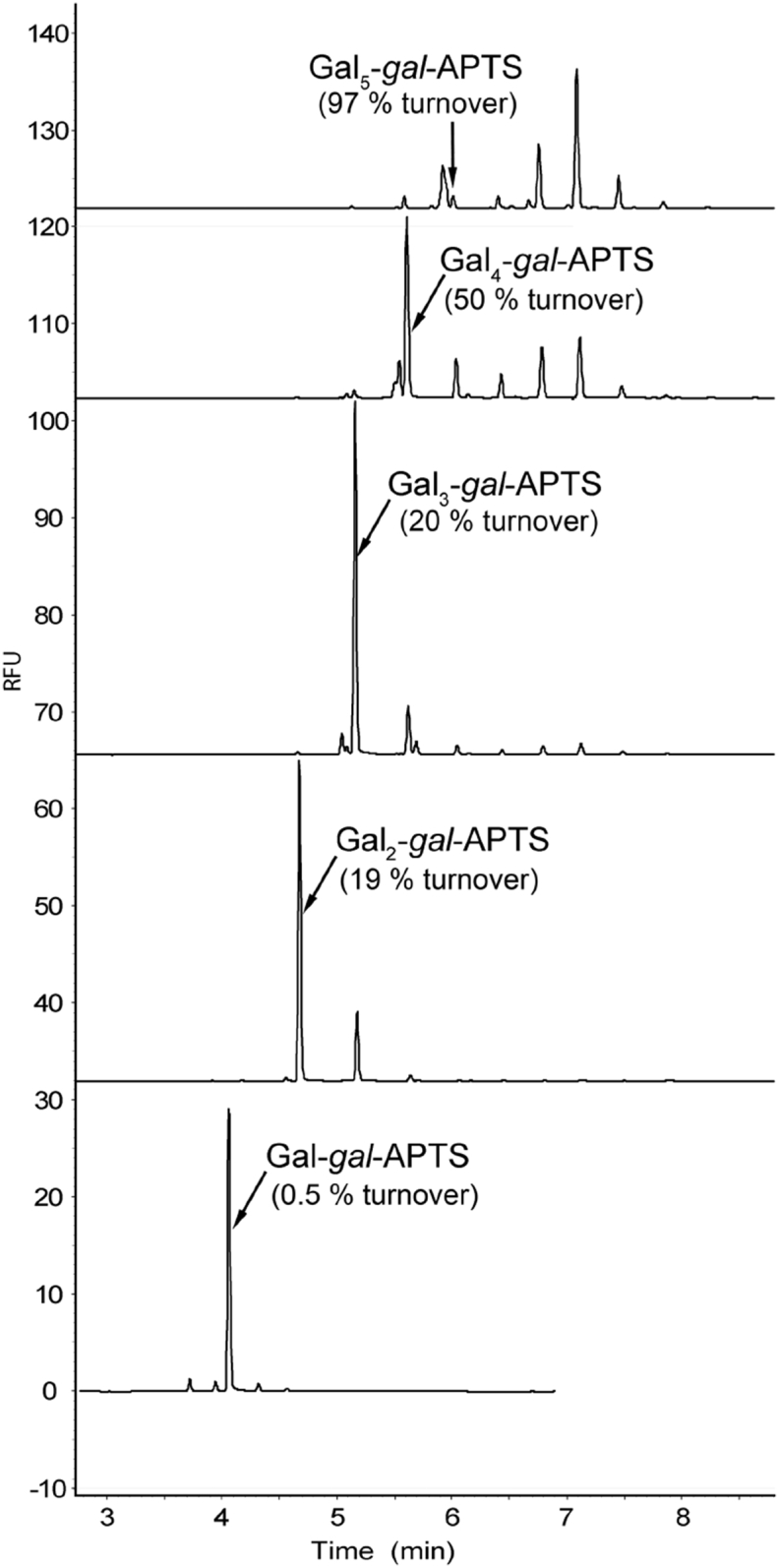
CE-LIF analyses of GalT assays with APTS-labelled β-(1 → 4)-*galacto*-oligosaccharides. Arrows show peak positions of acceptor substrates and values in parenthesis indicate percentage of their turnover in the corresponding assay. The galactosyltransferase reaction was performed for 4 h at 15 °C with 50 μg of *A. thaliana* microsomal protein in 0.5% Triton X-100 (detergent: protein ratio 5:1), 25 mM MES-KOH buffer pH 6.5 with 15 mM MnCl_2_, 50 μM APTS-labelled acceptors and 0.5 mM UDP-Gal.

In the first series of experiments with APTS-labelled galacto-oligosacharides, GalT assays were carried out with the *A. thaliana* enzyme preparation at 15 °C for 4 h. Under these conditions, formation of higher oligomers was readily monitored with the help of CE-LIF analysis (Fig. 5). Quantification of acceptor turnover was possible based on CE-LIF and the data are shown above corresponding electropherograms. The turnover increased with increasing acceptor galactan chain length, from a small but detectable extent in the case of Gal-β-(1 → 4)-*gal*-APTS (**Gal-*gal*-APTS**) to nearly complete consumption of the labelled acceptor substrate in case of **Gal**_**5**_**-*gal*-APTS**. Similar GalT assays with parent unlabelled *galacto*-oligosaccharides, followed by APTS labelling, revealed a small amount of polymerisation products only for β-(1 → 4)-*galacto*-pentaose and –hexaose, whereas chain elongation for β-(1 → 4)-*galacto*-biose, -triose, and –tetraose was undetectable by CE-LIF analysis (Fig. S6 in SI).

For comparison with previously published work [16], a second series of experiments was carried out with equivalent amounts of microsomal protein isolated from *V. radiata*. In this instance, after the same incubation time as for Arabidopsis microsomes (4 h), **Gal-*gal*-APTS** turnover gave rise to only trace quantities of two products. In contrast, polymerisation occurs much faster with **Gal_2_-*gal*-APTS** with about 50% turnover after 20 min incubation giving oligosaccharide products with up to 12 galactose residues (Fig. S7).

**Fig. 6:**
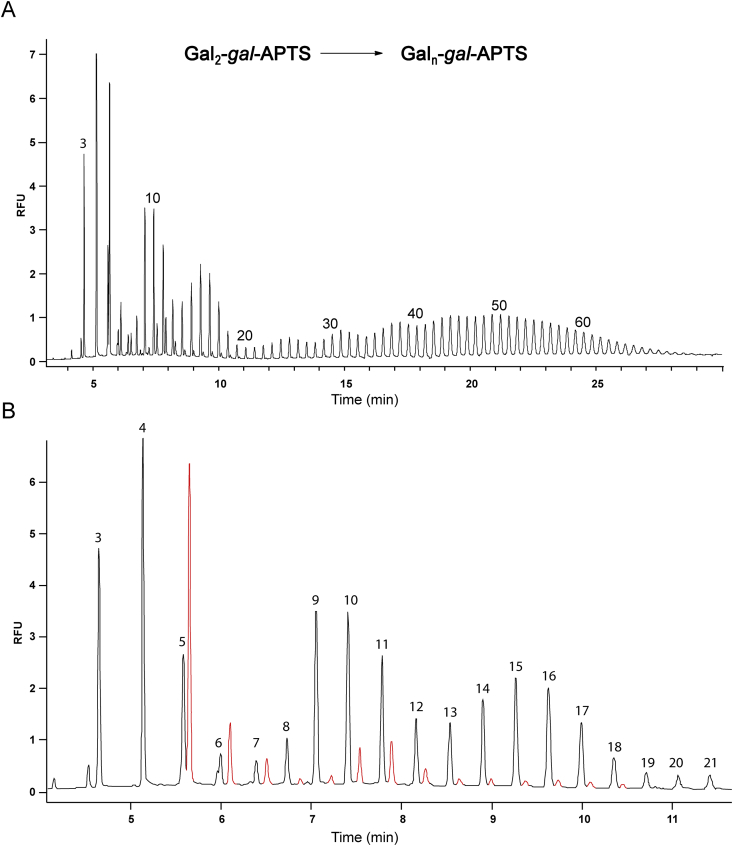
GalT assay with **Gal_2_-*gal*-APTS**. (A) CE-LIF analysis of reaction products after 17 h incubation; (B) Zoomed in view of electropherogram A. Estimated DPs (total number of Gal and *gal* residues) of products are shown above selected peaks. Peaks coloured red are likely to represent galactose-containing oligosaccharides with β-(1 → 3)- or β-(1 → 6)-linkages. The galactosyltransferase reaction was performed overnight at room temperature with 50 μg of Arabidopsis microsomal protein in 0.5% Triton X-100 (detergent/protein ratio 5:1), 25 mM MES-KOH buffer pH 6.5 with 15 mM MnCl_2_, 100 μM **Gal**_**2**_**-gal-APTS** and 1 mM UDP-Gal.

Finally, we examined the effect of prolonged action of Arabidopsis microsomes onto **Gal_3_-*gal*-APTS**. When the reaction was carried out overnight at 20 °C a wide range of products with DP reaching more than 60 was identified by CE-LIF (Fig. 6). Product peaks were regularly spaced, except at low retention time (<10 min) where additional set of signals were present (Fig. 6B, coloured red). The latter are likely to originate from products with β-(1 → 3) or β-(1 → 6)-linked galactose residues.

For the longer glycans produced, it appeared that the peak intensities had an oscillating characteristic, with an apparent series of overlapping distributions of peaks. While the biological significance of these observations remains to be clarified, the data may be consistent with the action of a series of polymerising β-(1 → 4)-GalTs with different acceptor chain length specificities.

Overall, these results support the finding that the APTS label in acceptor glycan substrates has a positive impact on Arabidopsis β-(1 → 4)-galactan:β-(1 → 4)-galactosyltransferase(s), which required further investigation.

### 3.6 The chemical structure of APTS contributes to the acceptor-enhancing effect

**Fig. 7:**
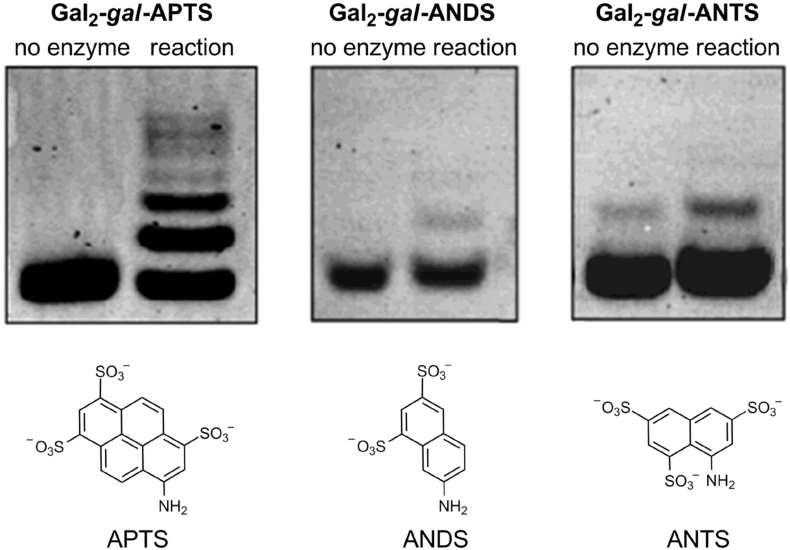
GalT assay with *A. thaliana* microsomes and **Gal**_**2**_**-*gal*-APTS**, **Gal**_**2**_**-*gal*-ANDS** and **Gal**_**2**_**-*gal*-ANTS** analysed by FACE as described in section 2.6. The reactions were performed overnight at 20 °C with 0.25 or 0.5 mM fluorescently-labelled galactotriose, 0.5 mM UDP-Gal and 100 μg of microsomal protein in 1.25% Triton X-100 (detergent/protein ratio 5:1), 25 mM Mes-KOH buffer pH 6.5 and 20 mM MnCl_2_.

The difference in GalT activities detected with APTS-labelled and unlabelled acceptors clearly indicates the positive effect of APTS on the ability of galactose-containing oligosaccharides to serve as substrates in β-galactan chain extension reactions. To gain insight into the influence of the chemical structure of APTS on APTS-labelled substrates, experiments were also performed with two other fluorescent labels, ANTS and ANDS, which are also used for fluorophore-assisted carbohydrate electrophoresis (FACE) [29], [35]. Galactotriose was fluorescently-labelled with ANDS and ANTS to give compounds similar to **Gal**_**2**_**-*gal*-APTS**. All three fluorescent derivatives were applied as substrates in parallel GalT assays, which were analysed by FACE (Fig. 7). With **Gal**_**2**_**-*gal*-APTS**, at least seven product bands were evident, which can be assigned to oligosaccharide products with DP 4–10. In contrast, only minor quantities of products arising from the addition of one or two galactose residues were detectable when either **Gal**_**2**_**-*gal*-ANDS** or **Gal**_**2**_**-*gal*-ANTS** were used.

These data demonstrate a clear and positive impact specifically of APTS label on galactosyltransferase activity. We note that the impact of a fluorescent label on glycan-protein binding selectivity has been observed elsewhere in glycoscience [36] and careful choice of fluorescent dye is necessary to achieve realistic results in fluorescence-based assays [37].

## 4 Conclusions

In this study we have demonstrated that readily accessible, APTS-labelled disaccharides are sufficient to serve as acceptor substrates for plant galactosyltransferases. The reductive amination procedure used to install the fluorophore into the substrate results in loss of the ring structure of the reducing terminal sugar unit, such that a single intact sugar ring is present, attached via an alditol tether to the aminopyrene fluorophore. The configuration of the alditol portion of the labelled acceptor, as well as the position of alditol galactosylation, substantially influence the ability of compounds to serve as Arabidopsis galactosyltransferase acceptor substrates. APTS-labelled disaccharides with a single terminal galactopyranose unit are chemically competent acceptors and are much more efficient than the corresponding unmodified parent disaccharides in plant galactosyltransferase assays. The lack of reactivity of unlabelled disaccharide acceptors suggests that the APTS label exhibits an unexpected reaction-promoting effect, one that is not evident for structurally similar sulfonated aromatic fluorophores ANDS and ANTS. Previous studies on the influence of the acceptor galactan chain length specificity of polymerising GalTs from *G. max* and *V. radiata* have revealed that at least a tetrasaccharide was required to prime polymerisation, with no measurable products detectable with shorter acceptors; maximum activity was achieved with acceptor galactans of seven galactose residues [16], [17]. In contrast to literature reports, where no turnover could be detected with 2-AB-labelled galactotriose acceptor [16], in our experiments conversion of even **Gal-*gal-*APTS** into longer oligomers was observable, albeit at low efficiency. However, when **Gal**_**2**_**-*gal*-APTS** was employed as an acceptor substrate with Arabidopsis microsomes, extensive elongation was observed, generating β-(1 → 4)-galactan chains running to beyond 60 galactose residues.

In summary, the data reported herein highlight the suitability of even very short glycan-APTS probes for assessing plant galactosyltransferase activities. In addition, while CE-LIF is established as a tool for glycan structural profiling, the potential of this approach for CAZyme profiling [38] is also evident from the present study.
