# Supplementary material for: The impact of aminopyrene trisulfonate (APTS) label in acceptor glycan substrates for profiling plant pectin β-galactosyltransferase activities
Source: Carbohydr Res. 2016 Oct 4;433:97–105. doi: 10.1016/j.carres.2016.07.017 (PMC5036537; doi:10.1016/j.carres.2016.07.017)
Supplement: Supplementary file 1 [file mmc1.docx]

**Supporting Information**

**The impact of aminopyrene trisulfonate (APTS) label in acceptor glycan substrates for profiling plant pectin β-galactosyltransferase activities**

Stephan Goetz, Martin Rejzek, Sergey A. Nepogodiev and Robert A. Field*

**List of Figures**

Fig. S1. Biochemical characterisation of the polymerising β‐(1→4)‐GalT.

Fig. S2 CE-LIF Analysis of the three APTS-labelled galactose-terminating disaccharide acceptors.

Fig. S3. Galactosyltransferase assay with Gal-β-(1→4)-*gal*-APTS as an acceptor analysed by CE-LIF.

Fig. S4. CE-LIF analysis of galactosyltransferase assays with unlabelled reducing sugars followed by post-reaction labelling.

Fig. S5. CE-LIF analysis of APTS-labelled galactooligosaccharides prepared by β-galactan hydrolysis.

Fig. S6. Comparison of GalT assays with APTS-labelled and unlabelled galacto-oligosaccharide acceptors.

Fig. S7. Galactosyltransferase assay with **Gal-*gal*-APTS** or **Gal_2_-*gal*-APTS** and microsomes isolated from *Vigna radiata*.

Table S1. ESI-MS data for APTS-labeled galactooligosaccharides

Fig. S8. ESI-MS (–) of Gal-β-(1→4)-*glc*-APTS.

Fig. S9. ESI-MS (–) of Gal-β-(1→6)-*glc*-APTS.

Fig. S10. ESI-MS (–) of Gal-β-(1→4)-*gal*-APTS.

Fig. S11. ESI-MS (–) of Gal-β-(1→4)-Gal-β-(1→4)-*gal*-APTS,

Fig. S12. ESI-MS (–) of Gal-β-(1→4)-[Gal-β-(1→4)]_2_-*gal*-APTS.

Fig. S13. ESI-MS (–) of Gal-β-(1→4)-[Gal-β-(1→4)_3_-*gal*-APTS.

Fig. S14. ESI-MS (–) of Gal-β-(1→4)-[Gal-β-(1→4)_4_-*gal*-APTS.


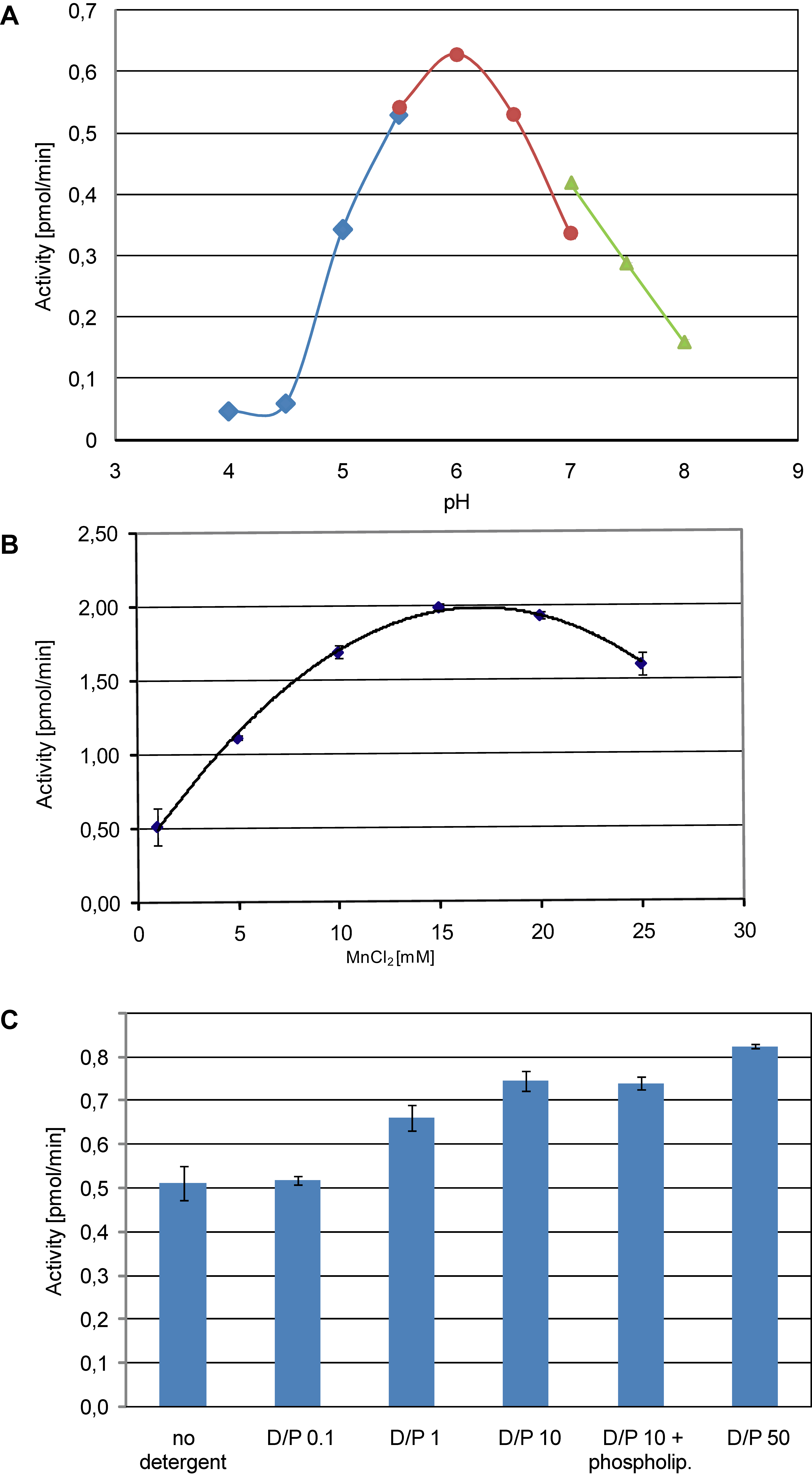


Fig. S1. Biochemical characterisation of the polymerising β‐(1→4)‐GalT. **A**, Effects of pH. Buffer concentration in the assay was 75 mM, (♦) acetate buffer, (●) MES‐KOH, (▲) Hepes‐KOH. **B**, Effect of MnCl_2_ concentration. **C,** Effect of detergent : protein (D/P) ratio. The detergent : protein ratio was calculated as (w/w) ratio using Triton X‐100 as detergent and microsomal protein. A D/P ratio of 0.1, 1.0, 10 and 50 equates 0.01 %, 0.1 %, 1.0 % and 5.0 % Triton X‐100 in the final reaction. All further reactions were performed with 25 μg of microsomal protein in 0.5 % Triton X‐100 (D/P = 5), 25 mM Mes‐KOH buffer pH 6.5 with 20 mM MnCl_2_, 100 μM **Gal_2_‐*gal*‐APTS** and 500 μM UDP‐Gal. The reaction time was 3 h at 15 °C. All data are the average of triplicate assays. The reactions were analysed by CE. Reaction rates were calculated by integration (corrected area percent) of the corresponding peaks for the acceptor **Gal_2_‐*gal*‐APTS** and the first product **Gal_3_‐*gal*‐APTS.**

Fig. S2 CE-LIF Analysis of the three APTS-labelled galactose-terminating disaccharide acceptors. Structures of these three compounds were confirmed by ESI-MS (negative mode) which revealed major peaks at *m/z* 309.9, 309.8 and 309.8, respectively, which correspond to [M-2H]^2-^ ion (calcd *m/z* 390.5).


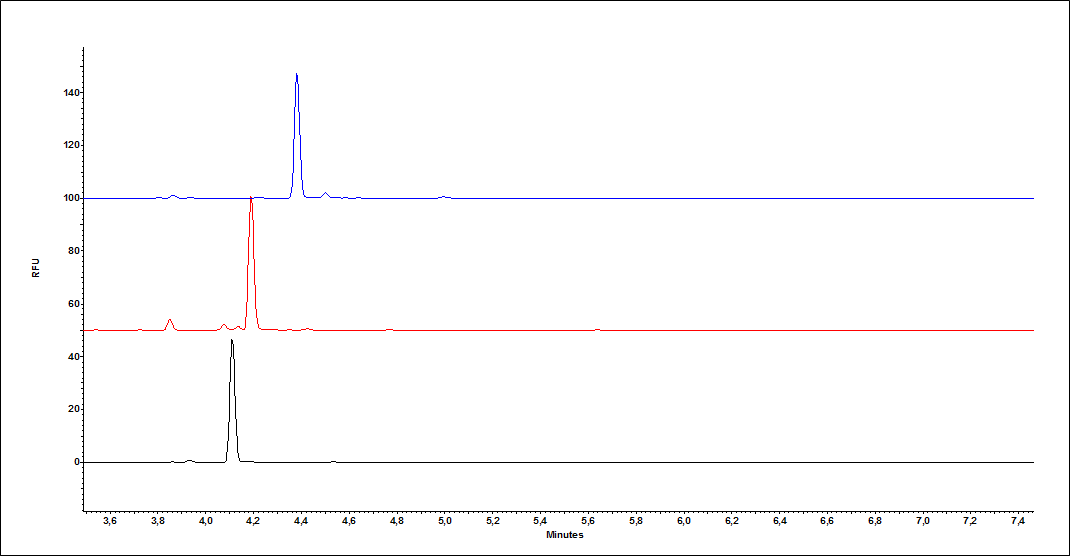


Gal-β-(1→6)-*gal*-APTS

Gal-β-(1→4)-*gal*-APTS

Gal-β-(1→4)-*glc*-APTS

Fig. S3. Galactosyltransferase assay with Gal-β-(1→4)-*gal*-APTS as an acceptor analysed by CE-LIF. **A**, blue: electropherogram of the Gal-β-(1→4)-*gal*-APTS; black: elongated products after incubation of Gal-β-(1→4)-*gal*-APTS with microsomes and UDP-Gal. **B**, blue and black: expanded versions of blue and black traces from A; green trace: digestion of the reaction mixture (black trace) with 2 μU exo-β-(1→4)-galactosidase (*Streptococcus pneumonia*e) for 24 h; DP marked below; red asterisk marks β-(1→4)-linked galacto-oligosaccharides. **C**: Further expanded section of the electropherogram from the reaction product (B). The galactosyltransferase reaction was performed for 5 days at 20 °C with 100 μg of microsomal protein in 1.25 % Triton X-100 (detergent/protein ratio = 5), 25 mM Mes-KOH buffer pH 6.5 with 15 mM MnCl_2_, 100 μM Gal-gal-APTS, 2.5 % glycerol and 0.5 mM UDP-Gal.


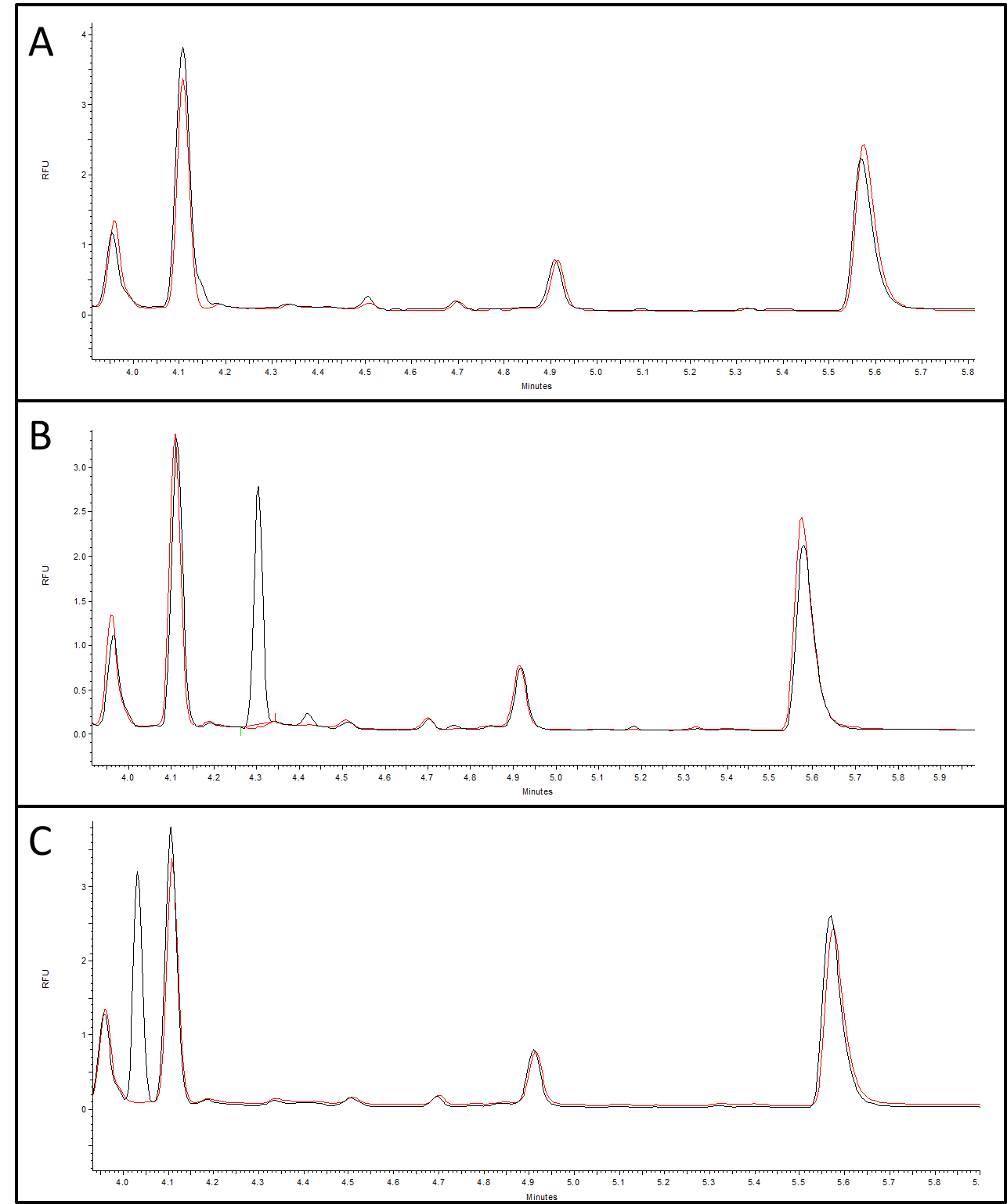


Gal-β-(1→4)-*glc*-APTS

Gal-β-(1→6)-*gal*-APTS

Gal-β-(1→4)-*gal*-APTS

Fig. S4. CE-LIF analysis of galactosyltransferase assays with unlabelled reducing sugars followed by post-reaction APTS labelling. Black traces resulted from CE-LIF analysis of reactions with acceptors while red traces are controls without acceptors. **A**, Gal-β-(1→4)-Gal; **B**, Gal-β-(1→6)-Gal; **C**, Gal-β-(1→4)-Glc. The galactosyltransferase reactions were performed for 2 days at 20 °C with 100 µg of microsomal protein in 1.25 % Triton X-100 (detergent : protein ratio 5:1), 25 mM Mes-KOH buffer pH 6.5 with 15 mM MnCl_2_ 250 µM unlabelled acceptor, 2.5 % glycerol and 0.5 mM UDP-Gal. APTS labelling was performed after completion of incubation.


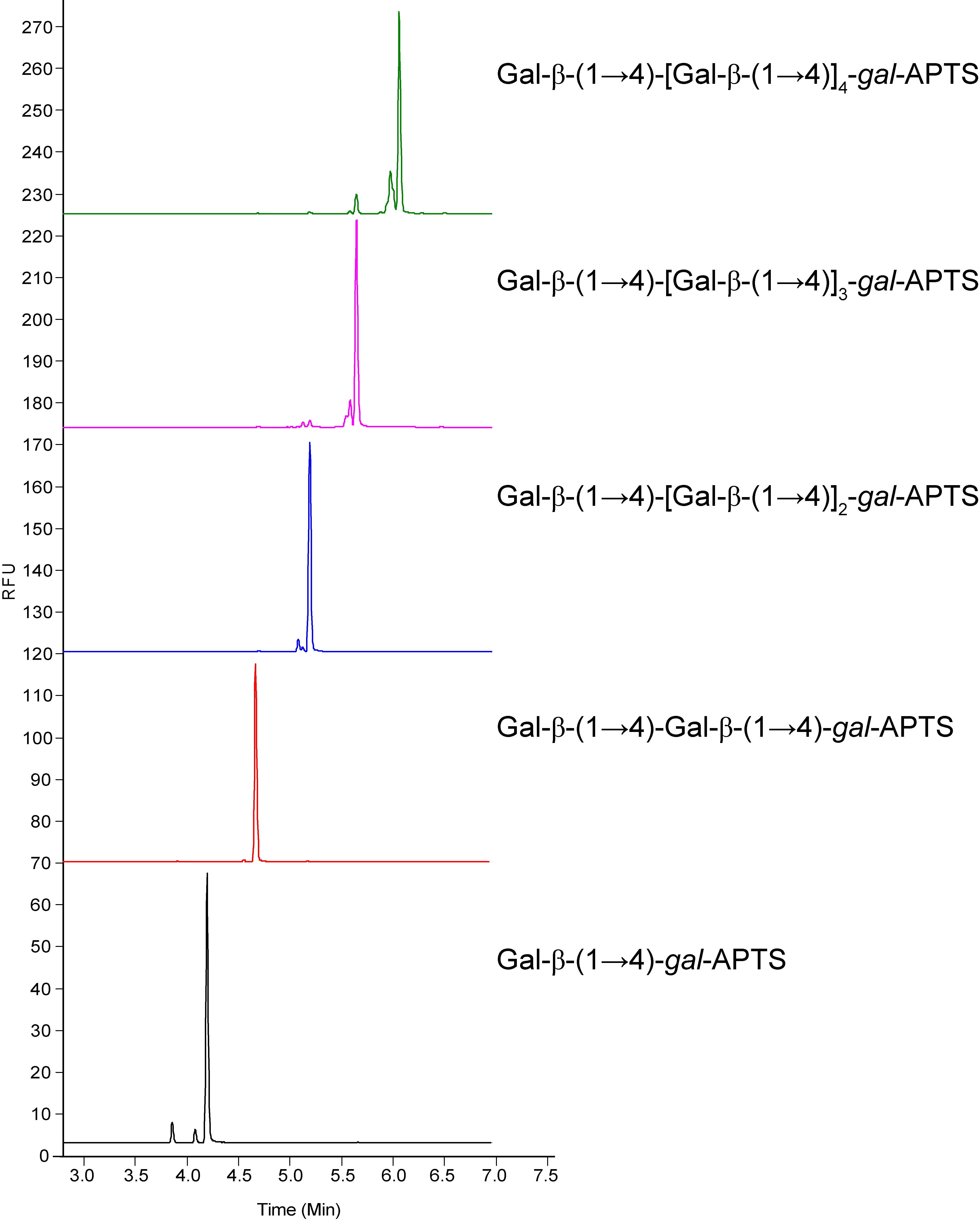


Fig. S5. CE-LIF analysis of APTS-labelled galactooligosaccharides prepared by β-galactan hydrolysis.


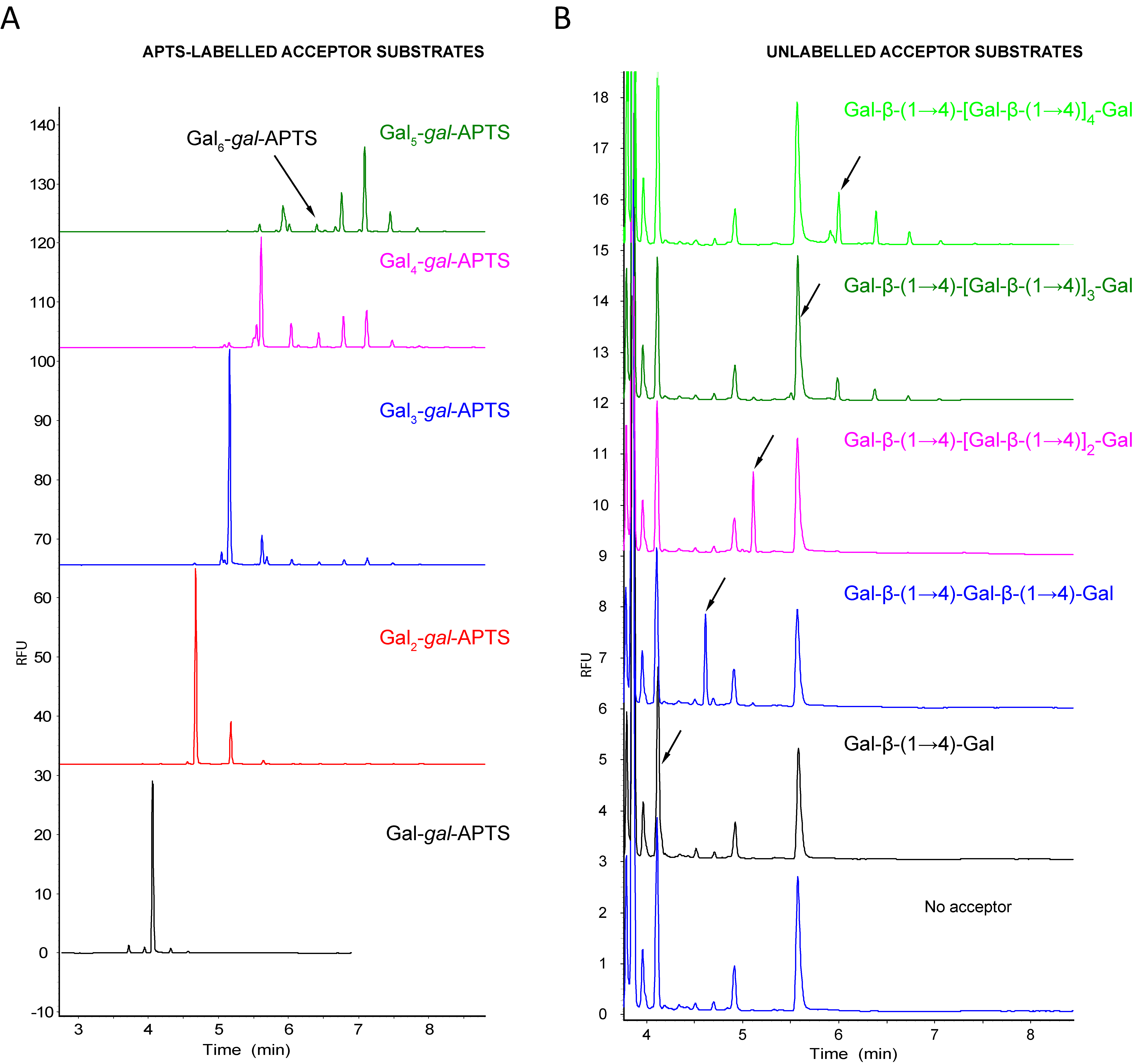


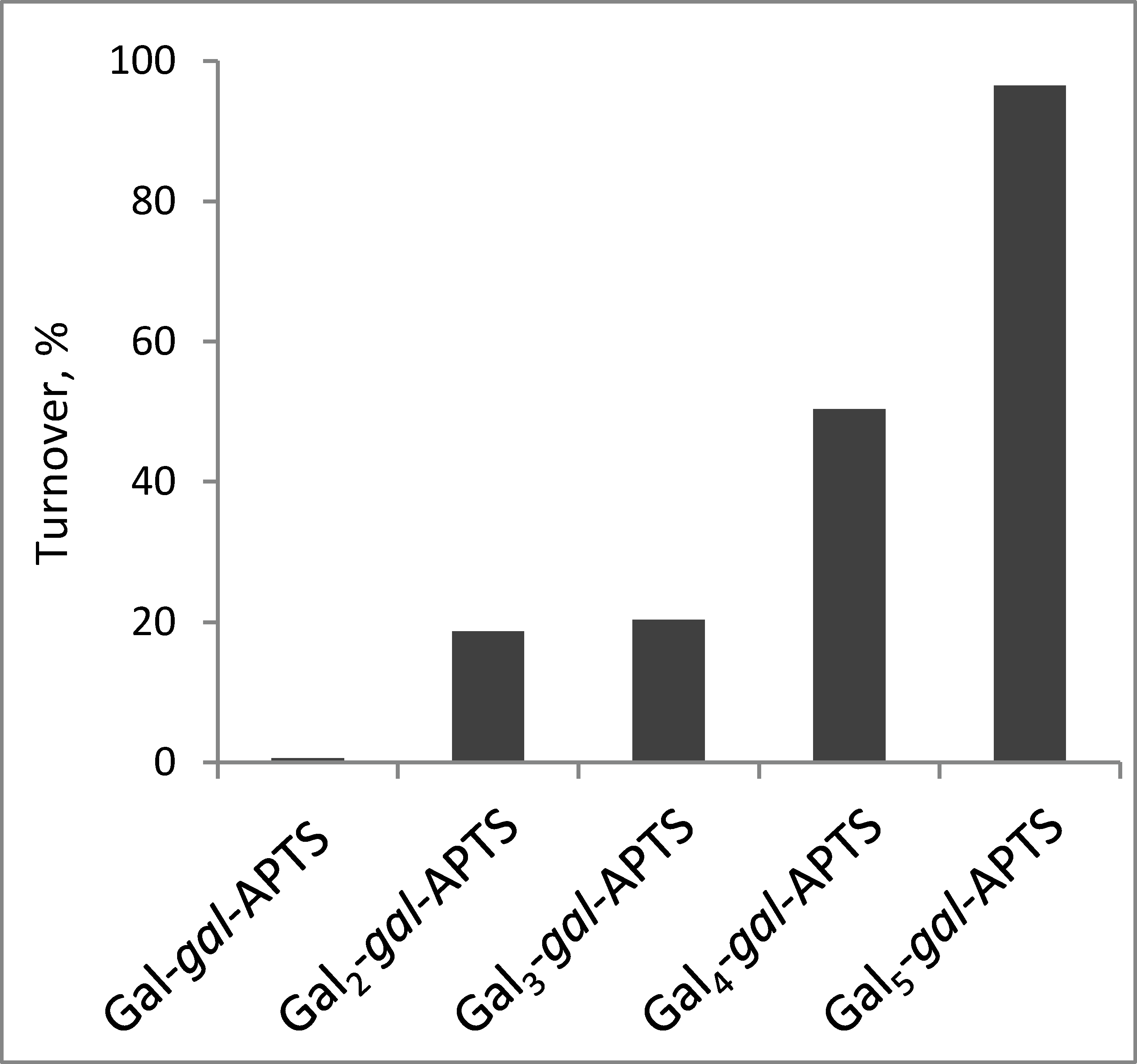


C

Fig. S6. Comparison of GalT assays with APTS-labelled and unlabelled galacto-oligosaccharide acceptors. **A**, CE-LIF analyses of reactions with APTS-labelled galacto-oligosaccharide acceptors, CE traces are labelled according to the substrate used. The galactosyltransferase reaction was performed for 4 h at 15 °C with 50 µg of microsomal protein in 0.5 % Triton X-100 (detergent : protein ratio 5:1), 25 mM Mes-KOH buffer pH 6.5 with 15 mM MnCl_2_, 50 μM APTS-labelled acceptors and 0.5 mM UDP-Gal. **B**, CE-LIF analyses of reaction with unlabelled galacto-oligosaccharides. Traces are labelled according to the substrate used and arrows indicate positions of peaks corresponding to substrates after APTS labelling. **C**, Quantitative analysis based on CE-LIF data. The reaction was performed over night at 20 °C with 100 μg of microsomal protein in 1.25% Triton X-100 (detergent : protein ratio 5:1), 25 mM Mes-KOH buffer pH 6.5 with 20 mM MnCl_2_, 0.25 mM acceptor and 0.5 mM UDP-Gal. Reactions were stopped by boiling followed by centrifugation. Aliquots of the supernatant were labelled with APTS.


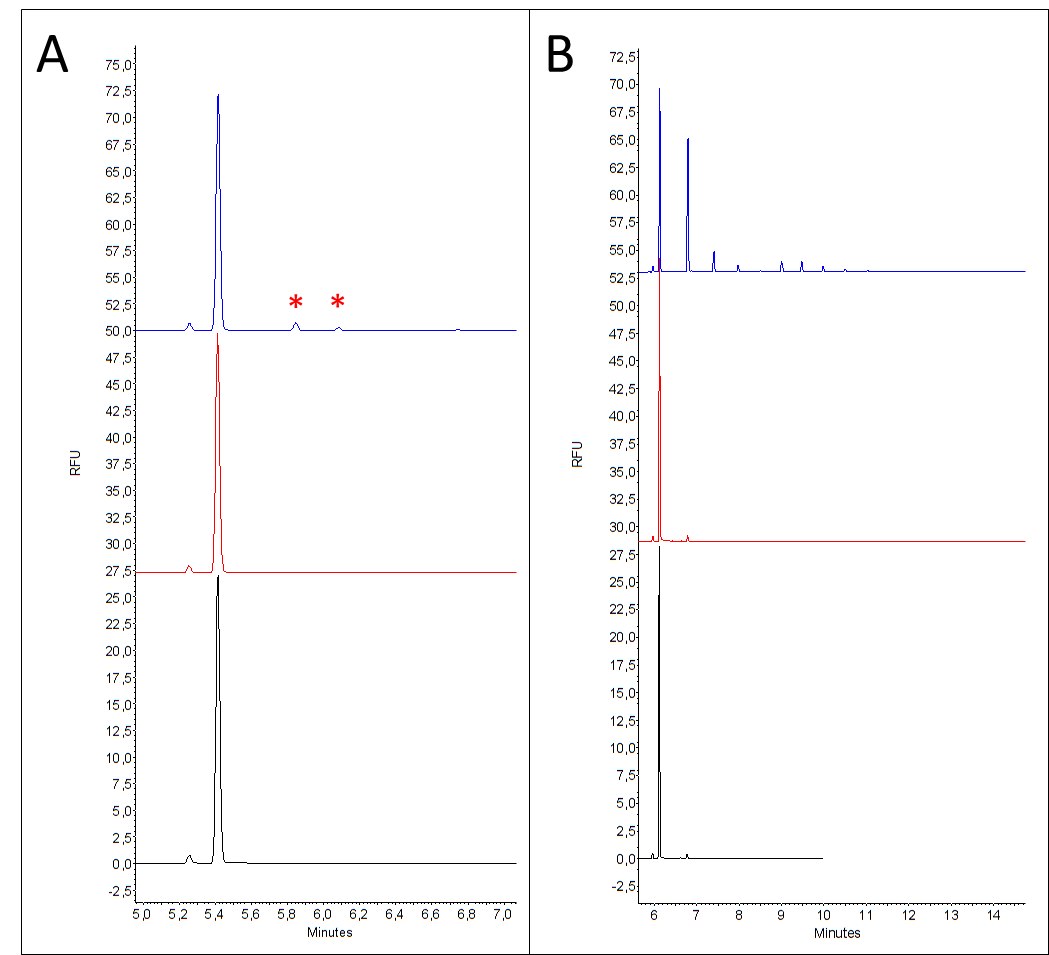


48%

34%

5%

2%

3%

3%

2%

2%

2%

**Gal_2_-*gal*-APTS** Reaction

Control – no UDP-Gal

**Gal_2_-*gal*-APTS**

**Gal-*gal*-APTS**

**Gal-*gal*-APTS** Reaction

Control – no UDP-Gal

Fig. S7. Galactosyltransferase assay with **Gal-*gal*-APTS** or **Gal_2_-*gal*-APTS** and microsomes isolated from *V. radiata*. **A**, CE-LIF analysis of the reaction with **Gal-*gal*-APTS**; **B**, CE-LIF analysis of the reaction with **Gal_2_-*gal*-APTS**. Red asterisks mark the positions of reaction products. For **Gal_2_-*gal*-APTS** reaction calculated peak areas are shown in percentage. The reactions were performed at 25 ˚C for 20 min in a total volume of 30 µl with 120 μg of microsomal protein, 160 mM sucrose, 20 mM NaF, 0.75 % Triton X-100, 40 mM MES-KOH buffer pH 6.5 with 25 mM MnCl_2_, 10 μM APTS-labelled acceptor and 2 mM UDP-Gal.

Table S1. ESI-MS data for APTS-labeled galactooligosaccharides*

| **Structure** |  | **Exact Mass** | **Calcd [M-2H]^2-^** | **Observed [M-2H]^2-^** |
| --- | --- | --- | --- | --- |
| Gal-β-(1→4)-*gal*-APTS | **Gal-*gal*-APTS** | 783.0809 | 390.5331 | 390.9 |
| Gal-β-(1→4)-Gal-β-(1→4)-*gal*-APTS | **Gal_2_-*gal*-APTS** | 943.1181 | 471.5596 | 472.0 |
| Gal-β-(1→4)-[Gal-β-(1→4)]_2_-*gal*-APTS | **Gal_3_-*gal*-APTS** | 1105.1709 | 552.5860 | 553.0 |
| Gal-β-(1→4)-[Gal-β-(1→4)]_3_-*gal*-APTS | **Gal_4_-*gal*-APTS** | 1267.2237 | 633.6124 | 634.0 |
| Gal-β-(1→4)-[Gal-β-(1→4)]_4_-*gal*-APTS | **Gal_5_-*gal*-APTS** | 1429.2765 | 714.6388 | 715.1 |

*ESI mass-spectra: Fig. S8-S14.

[M-2H]^2-^

Fig. S8. ESI-MS (–) of Gal-β-(1→4)-*glc*-APTS.

[M-2H]^2-^

Fig. S9. ESI-MS (–) of Gal-β-(1→6)-*glc*-APTS.

[M-2H]^2-^

Fig. S10. ESI-MS (–) of Gal-β-(1→4)-*gal*-APTS.

[M-2H]^2-^

Fig. S11. ESI-MS (–) of Gal-β-(1→4)-Gal-β-(1→4)-*gal*-APTS,

[M-2H]^2-^

Fig. S12. ESI-MS (–) of Gal-β-(1→4)-[Gal-β-(1→4)]_2_-*gal*-APTS.

[M-2H]^2-^

Fig. S13. ESI-MS (–) of Gal-β-(1→4)-[Gal-β-(1→4)_3_-*gal*-APTS.

Fig. S14. ESI-MS (–) of Gal-β-(1→4)-[Gal-β-(1→4)_4_-*gal*-APTS.

[M-2H]^2-^
